# A System of Surgery, Etc.

**Published:** 1859-09

**Authors:** 


ART. IX.—*A System of Surgery: Pathological, Diagnostic, Therapeutic, and Operative.* By S. D. GROSS, M.D., Professor of Surgery in the Jefferson Medical College of Philadelphia, etc. etc. 2 vols. 8vo., pp. 1162–1198. Philadelphia: Blanchard & Lea. 1859.

WE have simply time, in the present number, to chronicle the appearance of this long-announced and long-expected work, and to say that, in point of mechanical execution, it is by far the most beautiful production of the kind that has ever been issued on this side of the Atlantic. It is illustrated by nine hundred and thirty-six engravings, executed in the best style; and forms a more complete body of the art and science of surgery than is anywhere else to be found in the English language. The author has, in fact, not allowed anything to escape him. Nearly seven hundred pages are devoted to the consideration of the principles of surgery, while the remainder of the volumes is taken up with the discussion of the nature and treatment of the various injuries and external diseases incident to the human frame. A much larger space than is customary has been allotted to surgical pathology and surgical diagnosis. The diseases of the eye and ear, and even of the teeth and gums, usually excluded from treatises of this description, have received a full share of attention. Another valuable feature which we notice in the work is the frequent reference to the author's personal experience, now extending through a period of upward of thirty years.

